# A Forgotten Catheter for Two Decades: Catheter-Associated Tricuspid Endocarditis Managed With Percutaneous Debulking

**DOI:** 10.7759/cureus.93816

**Published:** 2025-10-04

**Authors:** Deepika Mannem, Peter Oro, Maryum Ali, Azka Khan, Yuxin Tian, Joseph Abdalla, Riley Smith, Mark Regala, Melina Aguinaga-Meza

**Affiliations:** 1 Department of Internal Medicine, Cleveland Clinic - South Pointe Hospital, Warrensville Heights, USA; 2 Department of Internal Medicine, Ohio University Heritage College of Osteopathic Medicine, Warrensville Heights, USA; 3 Department of Medicine, Markham Stouffville Hospital, Markham, CAN; 4 Department of Pulmonary/Critical Care, Cleveland Clinic - South Pointe Hospital, Warrensville Heights, USA; 5 Department of Cardiovascular Medicine, Cleveland Clinic - South Pointe Hospital, Warrensville Heights, USA

**Keywords:** angiovac debulking, catheter-associated endocarditis, catheter-associated infection, mssa bacteremia, mssa endocarditis, percutaneous debulking, prolonged antibiotic therapy, retained central venous catheter

## Abstract

We herein report a rare case of catheter-associated methicillin-susceptible *Staphylococcus aureus* (MSSA) endocarditis in a patient with a central venous access device retained for over 20 years. Diagnosis was delayed due to subtle clinical signs and limitations of initial imaging, but the patient was ultimately treated successfully with percutaneous debulking of the vegetation. This case highlights the risks associated with long-term catheter retention and underscores the importance of multidisciplinary care and innovative percutaneous therapies in high-risk patients.

## Introduction

Implantable venous access devices, such as central venous catheter devices, are commonly used for chemotherapy and long-term intravenous therapies [1]. The standard practice recommends removal once they are no longer clinically necessary [2]. Prolonged retention, especially beyond a decade, is rare but associated with serious complications, including infection, thrombosis, and endovascular injury [2,3]. Additionally, long-term retained catheters can become endothelialized or embedded within the vessel wall, forming fibrous sheaths that make percutaneous removal difficult and may necessitate open surgical extraction or vascular graft reconstruction in select cases [2].

Central venous catheters are a recognized risk factor for right-sided infective endocarditis (IE), particularly when the catheter tip lies near the right atrium [4]. Right-sided IE accounts for 5-10% of all IE cases and is frequently associated with indwelling devices [5]. Diagnosis of right-sided IE can be challenging as transthoracic echocardiography (TTE) has limited sensitivity for detecting right-sided vegetations, particularly in the presence of intracardiac devices [6]. Although transesophageal echocardiography (TEE) provides better visualization, it may still miss vegetations due to acoustic shadowing or suboptimal windows [6].

Surgical intervention is often required for persistent bacteremia, large vegetations, or embolic risk. However, many patients are poor surgical candidates due to age, frailty, or comorbidities. As such, percutaneous debulking of the right atrium is a promising procedure in selected cases.

We herein report a rare case of catheter-associated methicillin-susceptible *Staphylococcus aureus* (MSSA) endocarditis in a patient with a central venous access device retained for over 20 years. Diagnosis was delayed due to subtle clinical signs and limitations of initial imaging, but the patient was ultimately treated successfully with percutaneous debulking of the vegetation. This case highlights the risks associated with long-term catheter retention and underscores the importance of multidisciplinary care and innovative percutaneous therapies in high-risk patients.

## Case presentation

The patient is a woman in her 70s with a complex medical history, including stage 3a chronic kidney disease, hypothyroidism, hypertension, hyperlipidemia, obstructive sleep apnea, and depression. Her surgical history includes Roux-en-Y gastric bypass with multiple revisions and colon cancer complicated by hemicolectomy, followed by chemotherapy, for which a central venous access device was placed in the early 2000s. The catheter was never fully removed and remained in place with documented extension into the superior vena cava and right atrial junction. She also has a history of coagulase-negative *Staphylococcus *spine osteomyelitis and septic arthritis, treated in 2017 with a laminectomy, knee washout, and prolonged doxycycline course for the retained hardware and catheter fragments. She has a significant thrombotic history, including left subclavian and internal jugular deep vein thromboses, for which she was maintained on apixaban. She had multiple hospitalizations for fatigue, nausea, and a Corpak malfunction with recurrent MSSA bacteremia.

The patient presented to our institution in early May 2025 with chest pain, right arm discomfort, weakness, and a two-week history of subjective fevers and chills. The vital signs on initial presentation were temporal temperature of 100.6 °F, heart rate of 129 beats per minute (bpm), SpO_2_ of 93% on room air, and respirations of 16 per minute. She developed atrial fibrillation with rapid ventricular response and hypotension requiring digoxin loading, followed by amiodarone drip, beta-blockers, and norepinephrine. The patient was admitted to the ICU, placed on the sepsis care path, and started empirically on vancomycin and piperacillin-tazobactam.

Investigations, treatment, and outcome

On admission, the patient was febrile (38.1 °C), tachycardic (129 bpm), and mildly hypoxic (SpO₂ of 93% on room air). Laboratory evaluation revealed leukocytosis (white blood cell count: 18,000/μL), anemia (hemoglobin: 7.4 g/dL), thrombocytopenia (platelets: 76,000/μL), hyponatremia, and acute kidney injury (creatinine: 1.2 mg/dL) (Table 1). Inflammatory and cardiac biomarkers were markedly elevated, including NT-proBNP (17,898 pg/mL), C-reactive protein (16.6 mg/dL), and high-sensitivity troponin (125 → 116 → 111 ng/L), consistent with type II non-ST-elevation myocardial infarction (NSTEMI). EKG showed atrial fibrillation at a rate of 155 bpm. Blood and urine cultures grew MSSA (Table 2).

**Table 1:** Serial laboratory parameters during hospitalization Values represent key laboratory parameters tracked throughout hospitalization. H, high; L, low (based on institutional reference ranges). BUN, blood urea nitrogen; CO₂, carbon dioxide; eGFR, estimated glomerular filtration rate; WBC, white blood cell count; RBC, red blood cell count; MCV, mean corpuscular volume; MCH, mean corpuscular hemoglobin; MCHC, mean corpuscular hemoglobin concentration; RDW-CV, red cell distribution width–coefficient of variation; MPV, mean platelet volume; Neut%, neutrophil percentage; ANC, absolute neutrophil count; Lymph%, lymphocyte percentage; Abs Lymph, absolute lymphocyte count; CRP, C-reactive protein; NT-proBNP, N-terminal pro–B-type natriuretic peptide; hsTnT, high-sensitivity troponin T; mmol/L, millimoles per liter; mg/dL, milligrams per deciliter; pg/mL, picograms per milliliter; ng/L, nanograms per liter; k/µL, thousands per microliter; m/µL, millions per microliter; mL/min/1.73 m², milliliters per minute per 1.73 square meters of body surface area. *Reference ranges not provided by reporting institution.

Laboratory Test	Day 1	Day 9 (Pre-procedure)	Day 10 (Post AngioVac)	Day 12	Day 18 (Discharge)	Reference Range
BUN (mg/dL)	40 (H)	64 (H)	60 (H)	66 (H)	55 (H)	7-21 mg/dL
Creatinine (mg/dL)	1.20 (H)	2.45 (H)	2.35 (H)	2.11 (H)	1.46 (H)	0.58-0.96 mg/dL
Sodium (mmol/L)	131 (L)	129 (L)	130 (L)	130 (L)	136	136-144 mmol/L
Potassium (mmol/L)	4.2	4.1	4.0	4.6	4.0	3.7-5.1 mmol/L
Chloride (mmol/L)	99	96 (L)	96 (L)	99	101	98-107 mmol/L
CO_2_ (mmol/L)	24	19 (L)	18 (L)	18 (L)	23	22-30 mmol/L
Anion Gap (mmol/L)	8	14	16 (H)	13	12	8-15 mmol/L
Calcium (mmol/L)	7.9 (L)	7.9 (L)	7.5 (L)	7.6 (L)	8.2 (L)	8.5-10.2 mg/dL
eGFR (mL/min/1.73 m²)	48 (L)	20 (L)	22 (L)	24 (L)	38 (L)	>=60 mL/min/1.73 m²
WBC (k/uL)	18.06 (H)	14.43 (H)	23.89 (H)	15.40 (H)	8.42	3.70-11.00 k/uL
RBC (m/uL)	2.38 (L)	2.72 (L)	3.67 (L)	3.00 (L)	2.90 (L)	3.90-5.20 m/uL
Hemoglobin (g/dL)	7.4 (L)	8.0 (L)	10.8 (L)	9.0 (L)	9.0 (L)	11.5-15.5 g/dL
Hematocrit (%)	21.6 (L)	23.0 (L)	32.5 (L)	26.8 (L)	27.7 (L)	36.0-46.0%
MCV (fL)	90.8	84.6	88.6	89.3	95.5	80.0-100.0 fL
MCH (pg)	31.1	29.4	29.4	30.0	31.0	26.0-34.0 pg
MCHC (g/dL)	34.3	34.8	33.2	33.6	32.5	30.5-36.0 g/dL
RDW-CV (%)	19.8 (H)	21.3 (H)	19.6 (H)	20.0 (H)	18.8 (H)	11.5-15.0%
Platelet Count (k/uL)	76 (L)	402 (H)	351	358	397	150-400 k/uL
MPV (fL)	-	11.7	12.2	12.1	10.6	9.0-12.7 fL
Neut%	93.6	-	-	-	-	*
Abs Neut (ANC) (k/uL)	16.90 (H)	-	-	-	-	1.45-7.50 k/uL
Lymph%	2.5	-	-	-	-	*
Abs Lymph (k/uL)	0.46 (L)	-	-	-	-	1.00-4.00 k/uL
Lactate (mmol/L)	1.7	0.7	1.5	-	-	0.5-2.2 mmol/L
CRP (mg/dL)	16.6 (H)		2.1 (H)			<0.9 mg/dL
UltraSens C-Reactive Protein (mg/L)	65.2 (H)	-	-	-	-	<3.1 mg/L
NT Pro BNP (pg/mL)	17,898 (H)	-	-	-	-	<125 pg/mL
TNT High Sensitivity (ng/L)	111 (H)	-	-	-	-	<12 ng/L
	116 (H)	-	-	-	-	<12 ng/L
	125 (H)	-	-	-	-	<12 ng/L

**Table 2:** Microbiology These are the respective culture and smear results obtained throughout the patient’s hospital course. ^a^Obtained from the left lung pleural cavity thoracentesis on hospital admission day four. ^b^Heart tissue sample obtained during the AngioVac procedure on hospital admission day nine. MSSA, methicillin-sensitive Staphylococcus aureus; PMNs, polymorphonuclear leukocytes; NGTD, no growth to date; AngioVac, AngioVac Aspiration System (percutaneous vacuum-assisted thrombectomy device)

Day of Hospital Course	Culture	Smear	Source	Reference Range
Day 1	Staph aureus (MSSA)	Gram-positive cocci in clusters	Blood	Reference range for microbiological cultures: no growth or no organisms seen on Gram stain. The presence of PMNs may indicate inflammation, but is not specific to infection. Growth of bacteria or fungi was considered a positive culture result.
Day 4	Staph hominis	Gram-positive cocci in clusters	Blood	
Day 4	No growth	No organisms seen, many PMNs	Pleural Cavity^a^	
Day 9 (Pre-procedure)	Rare Staph aureus	No organisms seen, many PMNs	Heart Tissue^b^	
Day 10 (Post AngioVac)	Staph aureus (MSSA)	Gram-positive cocci in clusters	Blood	
Day 12	NGTD x 5 days		Blood	

**Figure 1:**
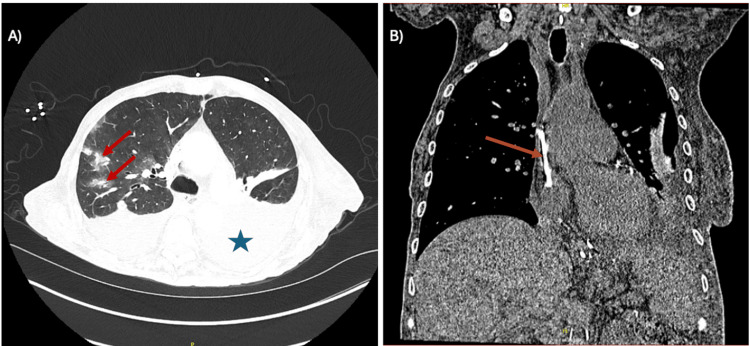
CT imaging of bilateral septic emboli and the retained intravascular catheter (A) Axial chest CT demonstrating bilateral multifocal ground-glass opacities and nodular consolidations, most prominent in the right lung (red arrows), consistent with septic emboli. Also noted are bilateral pleural effusions, more pronounced on the left (blue star). (B) Retained catheter fragment extending from the left innominate vein to the cavoatrial junction (orange arrow).

MRI of the thoracic spine and right shoulder showed only degenerative changes, without evidence of osteomyelitis or septic arthritis. Chest computed tomography demonstrated large left-sided and moderate right-sided pleural effusions, bilateral consolidations, multifocal ground-glass opacities, and nodular densities consistent with septic emboli. A retained catheter fragment was visualized extending from the left innominate vein to the cavoatrial junction (Figure 1).

Point-of-care ultrasound (POCUS) revealed normal biventricular function, a small pericardial effusion without tamponade physiology, and a collapsible inferior vena cava.

Left-sided thoracentesis yielded 1.2 L of amber-colored fluid. Pleural fluid analysis was consistent with a transudate, showing 15,000 RBCs/μL, 1,031 nucleated cells (65% neutrophils), LDH of 106 U/L, pH of 7.9, and protein of 1.7 g/dL. Cytology was negative for malignancy (Table 3) [7].

**Table 3:** Left lung pleural cavity thoracentesis results H, high; L, low (based on institutional reference ranges). Values represent the thoracentesis results after the left lung pleural cavity thoracentesis was performed on hospital day four. LDH, lactate dehydrogenase; BF, body fluid; RBC, red blood cell count; BCX, body fluid culture; pH, potential of hydrogen (acidity/alkalinity); Neut%, neutrophil percentage; Lymph%, lymphocyte percentage; Mono%, monocyte percentage; Meso%, mesothelial cell percentage; AFB, acid-fast bacilli; U/L, units per liter; g/dL, grams per deciliter; mL, milliliters *Reference ranges not provided by reporting institution. Interpreted per Light’s Criteria A, total protein, and fluid-to-serum protein ratio were used to classify effusions. An exudate was defined as body fluid total protein ≥ 3.0 g/dL or a fluid-to-serum total protein ratio ≥ 0.5. A transudate was defined as total protein < 3.0 g/dL and a fluid-to-serum protein ratio < 0.5 [7].

Reference ranges	Day 4	Reference Range
LDH, serum (U/L)	206	135-214 U/L A
LDH, body fluid	106	Interpreted per Light’s Criteria A
Protein, serum (g/dL)	4.3	6.3-8.0 g/dL A
Protein, body fluid	1.7	Interpreted per Light’s Criteria A
pH, body fluid	7.9	-
Color, BF	Amber	Yellow
Clarity, BF	Slightly cloudy	Clear
Amount (mL)	1200	-
RBC, body fluid	15,000 (H)	<2,000/uL
Total nucleated cells, BF	1,031 (H)	<1,000/uL
BCX, body fluid	No growth	No growth
Cytology	Negative for malignant cells. Acute and chronic inflammation	No malignant cells identified
Diff total body fluid	100	Cells counted
Neut%, BF	65 (H)	0-1%
Lymph%, BF	19	18-36%
Mono%, BF (%)	13	*
Meso%, BF	3 (H)	0-2%
AFB culture and stain	No acid-fast bacilli isolated after 21 days	No growth or organism seen
Fungal culture	No fungus isolated after 21 days	No growth or organism seen

**Figure 2:**
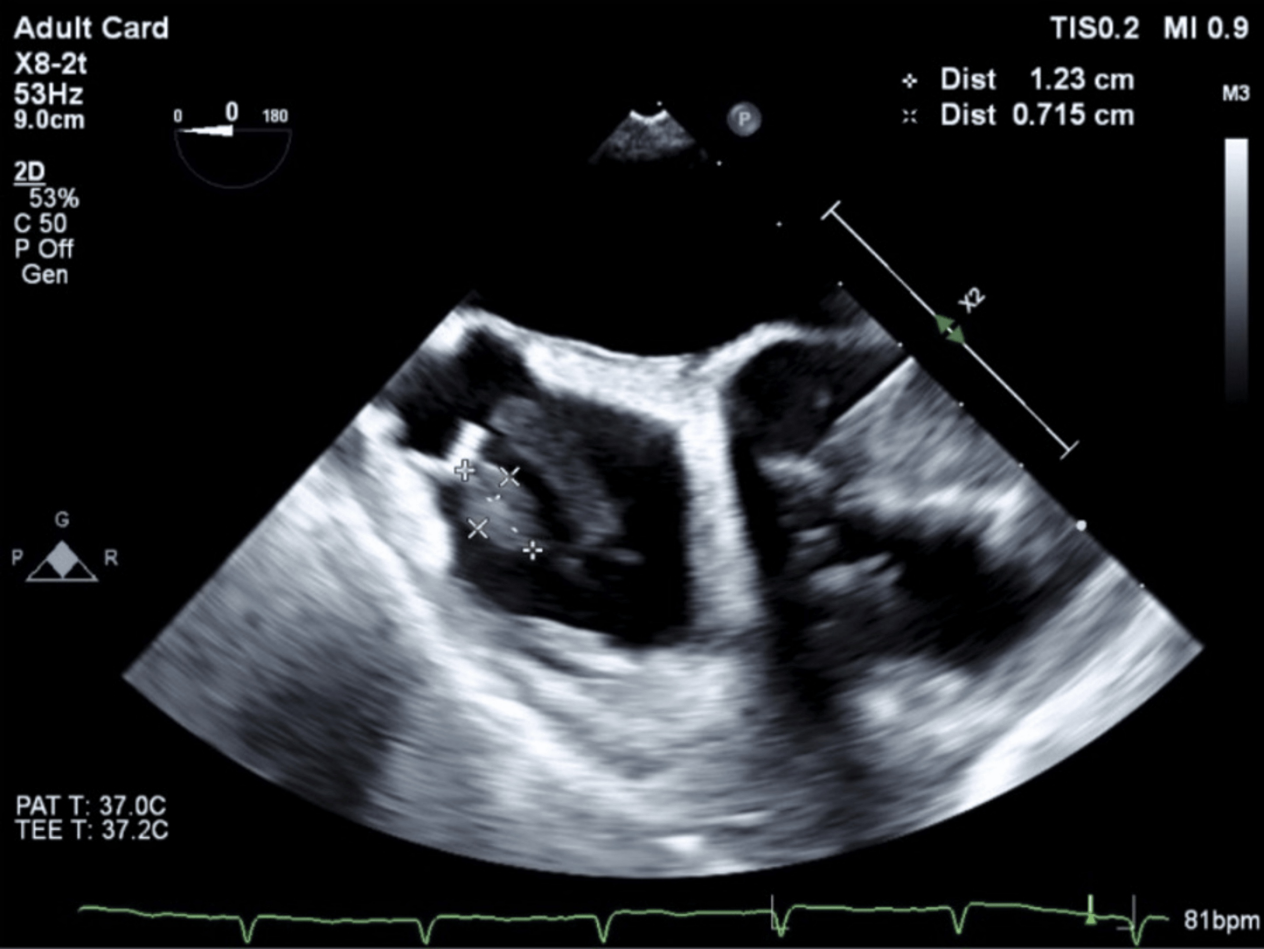
Transesophageal echocardiogram (mid-esophageal view) A large, mobile right atrial mass is seen measuring approximately 1.23 × 0.715 cm, consistent with vegetation attached to the catheter tip at the superior vena cava right atrial junction.

Given persistent bacteremia and the retained catheter fragment, TEE was performed, which revealed multiple large, mobile vegetations attached to the catheter tip at the superior vena cava-right atrial junction (the largest measuring 2.2 × 0.75 cm), along with a 1.6 cm vegetation on the anterior tricuspid leaflet and trace tricuspid regurgitation (Figure 2). Agitated saline contrast confirmed the presence of a patent foramen ovale (PFO).

The patient was initially started on empiric IV vancomycin and piperacillin-tazobactam for presumed sepsis related to a chronically retained catheter. After blood cultures grew MSSA, antibiotics were narrowed to cefazolin. Persistent bacteremia despite appropriate therapy raised concern for an endovascular source.

During her hospitalization, she also developed new-onset atrial fibrillation with RVR, which was managed with digoxin loading and an amiodarone drip. A right proximal femoral DVT was identified via ultrasound, and anticoagulation was initiated with a heparin drip.

**Figure 3:**
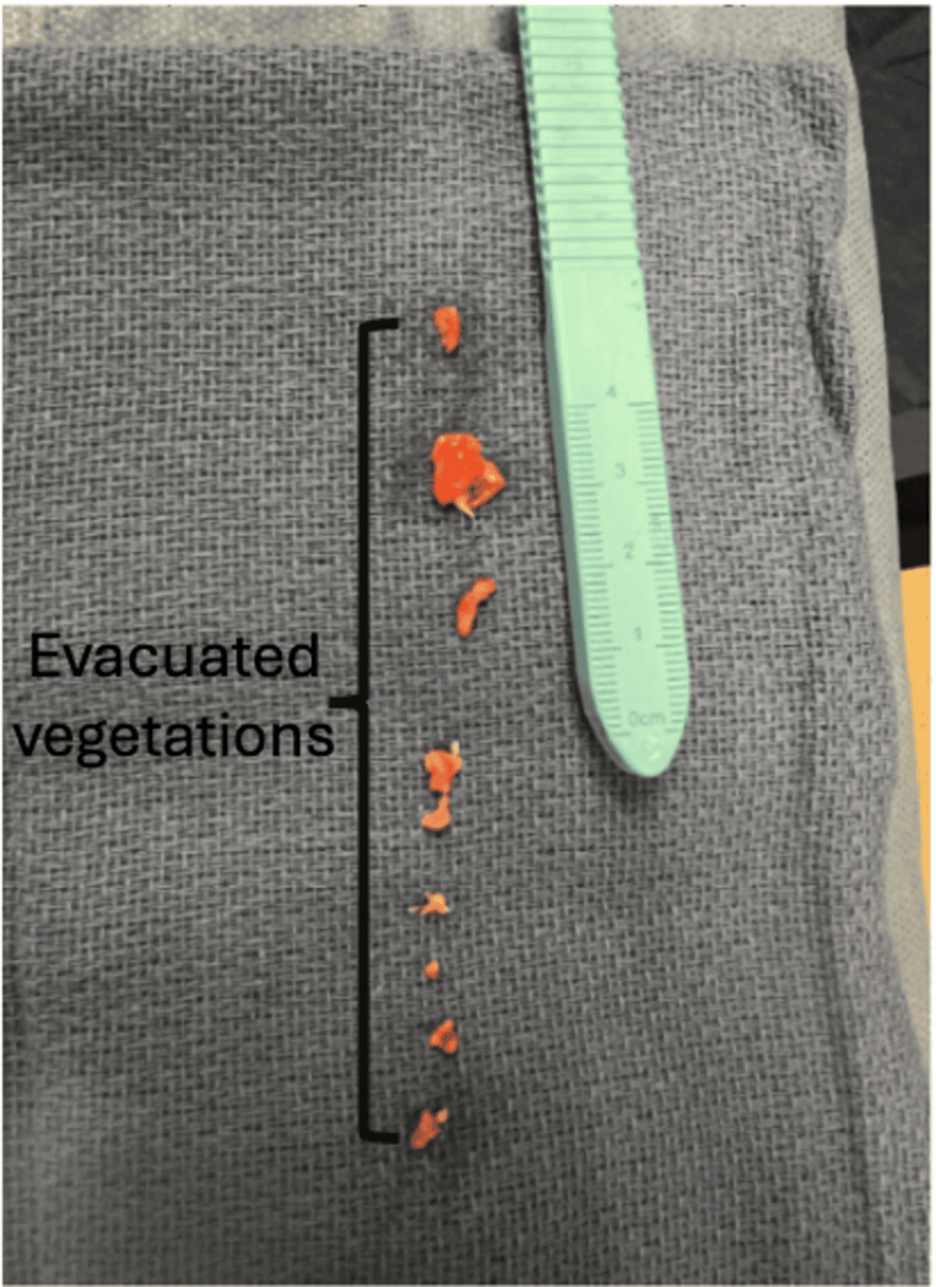
Evacuated vegetations

Following the discovery of a large tricuspid valve vegetation, surgical consultation was obtained. However, the patient was deemed a poor candidate for operative intervention due to frailty, chronic kidney disease, prior abdominal surgeries, and hemodynamic instability. She was referred to interventional cardiology, and percutaneous aspiration of the right atrial vegetation was performed using the AngioVac system (Figure 3, Table 4).

**Table 4:** Surgical pathology This is the result of the surgical pathology from the AngioVac procedure performed on hospital admission day nine. A Movat stain was performed for visualization and showed acute fibrinous vegetation with numerous polymorphonuclear leukocytes and bacterial colonies. Microorganisms were Gram variable on Gram stain. They were visible on Grocott’s methenamine silver (GMS) stain but not periodic acid-Schiff (PAS) stain. No fungal organisms were identified. There was also no valvular tissue present in the sample.

Surgical Pathology	Day 9
Source	Removed vegetation from the heart
Result	Acute vegetation with Gram-positive cocci

Bilateral femoral venous access was obtained, and intracardiac echocardiography (ICE) confirmed a large vegetation adherent to the catheter tip. The vegetation was successfully aspirated under ICE and fluoroscopic guidance, with post-procedure imaging confirming complete removal and no pericardial effusion.

Given prior TEE findings suggestive of a PFO, ICE-guided interrogation of the interatrial septum was performed. A catheter and wire entered the foramen ovale tunnel, but passage into the left atrium was unsuccessful. A bubble study revealed no right-to-left shunt, and the PFO was presumed to be functionally closed.

Following the procedure, blood cultures gradually cleared. Surgical pathology showed acute vegetation with Gram-positive cocci (Table 4). Given sustained MSSA sensitivity and clinical improvement, antibiotics were transitioned from cefazolin to intravenous oxacillin to complete a prolonged treatment course.

The patient's condition improved after treatment, and the patient was ultimately discharged to a skilled nursing facility with a tunneled catheter for completion of intravenous oxacillin therapy. Additional medications at discharge included amiodarone, hydroxyzine, and sublingual hyoscyamine.

## Discussion

Central venous catheter devices are essential for delivering long-term intravenous therapies; however, complications, although rare, can be severe. One particularly uncommon complication is the retention of catheter fragments following attempted removal, which substantially increases risks for serious clinical sequelae, including thrombosis, infection, and IE. While literature reports incidences ranging between 0.4% and 2.2%, the uniqueness of our case lies in the extraordinary indwelling duration (>20 years) of the catheter fragment, substantially exceeding typical durations previously described [8,9].

Notably, retained catheter fragments complicated specifically by large right-sided IE vegetations remain scarcely documented. In our patient, the retained catheter fragment significantly contributed to persistent MSSA bacteremia, which ultimately resulted in extensive tricuspid valve vegetations and septic pulmonary embolization. Furthermore, our experience highlights a critical diagnostic pitfall: despite large vegetations, initial TTE failed to visualize these lesions clearly, emphasizing the indispensable role of early TEE in similar complex presentations. Although TEE’s superior sensitivity (approaching 90-100%) compared to TTE (approximately 40-63%) is known, the severity and extent of IE missed initially by TTE in this scenario underscores the necessity of a high diagnostic vigilance when retained intravascular fragments are present [10].

A notable contribution of our case is the detailed clinical decision-making surrounding prolonged antibiotic therapy. While standard recommendations advise a minimum antibiotic duration of four to six weeks in IE, explicit guidelines for antibiotic treatment duration in patients with extensively retained catheters and persistent bacteremia remain insufficiently detailed [11]. Our patient required prolonged therapy with vancomycin and later cefazolin due to persistent positive cultures. This clinical experience underscores the importance of individualized antibiotic strategies in managing IE complicated by retained intravascular devices, particularly when initial antibiotic courses fail to achieve microbial clearance.

Surgical removal of infected retained catheter fragments and associated vegetations is generally indicated by the presence of persistent bacteremia, recurrent embolization, progressive valve dysfunction, heart failure, or large vegetation burden (>1-2 cm) despite appropriate antibiotic therapy [12]. Although our patient clearly met multiple surgical indications - including persistent bacteremia, multiple septic emboli, and large tricuspid valve vegetations (the largest measuring 2.2 cm) - the case illustrates a scenario where surgery is prohibitive. Multiple patient-specific factors - including severe malnutrition, advanced age, significant frailty, chronic kidney disease, extensive prior surgical interventions (multiple abdominal surgeries, including Roux-en-Y gastric bypass revisions), recent thrombotic events, and critical hemodynamic instability - precluded traditional surgical options, underscoring the critical importance of patient-tailored approaches in high-risk clinical scenarios.

One of the most novel aspects of our case is the successful use of the AngioVac percutaneous debulking system, employed effectively as a minimally invasive alternative to traditional surgery in a profoundly high-risk patient. While the AngioVac system has previously demonstrated effectiveness in the removal of right-sided vegetations, its successful application in a patient as medically compromised as ours significantly expands the clinical boundaries for percutaneous interventions [12]. Thus, our experience provides meaningful practical evidence supporting percutaneous mechanical debulking as a viable alternative in high-risk patients contraindicated for open cardiac surgery.

Optimal timing for central venous catheter removal to avoid severe complications, such as retention, remains unclear, though literature increasingly supports proactive catheter removal once no longer clinically necessary, especially after indwelling durations exceeding three to four years. Our experience strongly advocates for the proactive management and timely removal of central venous catheters, when indicated, to mitigate risks associated with excessively prolonged retention.

Finally, our case highlights a critical diagnostic consideration: differentiating true retained catheter fragments from calcified fibroblastic sheaths, as described by Baciarello et al. [13]. Fibroblastic sheaths mimicking retained catheter fragments on imaging studies pose significant risks for unnecessary interventions. Meticulous procedural documentation and advanced imaging, including three-dimensional computed tomography, provide crucial diagnostic clarity in these complex scenarios, preventing unnecessary invasive procedures.

## Conclusions

This unique clinical experience highlights significant learning points related to prolonged catheter retention, diagnostic pitfalls, therapeutic challenges, and innovative minimally invasive interventions. Retained central venous catheters, even decades old, can become overlooked sources of life-threatening infection; they should not be dismissed in the differential diagnosis of persistent bacteremia. In patients with intracardiac devices or hardware, transthoracic echocardiography may be falsely reassuring; early use of transesophageal echocardiography is essential. This case highlights the expanding role of AngioVac debulking as a lifesaving, minimally invasive alternative to surgery in critically ill patients with right-sided endocarditis who are not surgical candidates. Our findings underscore the importance of individualized antibiotic management, proactive catheter removal, careful surgical evaluation, and appropriate percutaneous interventions in complex clinical scenarios involving retained catheter fragments and infective endocarditis.
